# Induced Nematic Phase of New Synthesized Laterally Fluorinated Azo/Ester Derivatives

**DOI:** 10.3390/molecules26154546

**Published:** 2021-07-28

**Authors:** Fowzia S. Alamro, Hoda A. Ahmed, Mohamed A. El-Atawy, Salma A. Al-Zahrani, Alaa Z. Omar

**Affiliations:** 1 Department of Chemistry, College of Science, Princess Nourah bint Abdulrahman University, Riyadh 11671, Saudi Arabia; fsalamro@pnu.edu.sa; 2 Department of Chemistry, Faculty of Science, Cairo University, Cairo 12613, Egypt; 3 Chemistry Department, Faculty of Science, Taibah University, Yanbu 46423, Saudi Arabia; 4 Chemistry Department, Faculty of Science, Alexandria University, P.O. Box 426 Ibrahemia, Alexandria 21321, Egypt; alaazaki@alexu.edu.eg; 5 Department of Chemistry, College of Sciences, University of Ha’il, Ha’il 2440, Saudi Arabia; s.alzahrane@uoh.edu.sa

**Keywords:** phase behaviour, lateral fluorine, induced nematic phase, optimize structure, DFT

## Abstract

A new series of laterally fluorinated mesomorphic compounds, namely 2-fluoro-4-((4-(alkyloxy)phenyl)diazenyl)phenyl 4-substitutedbenzoate (**In_x_**) were prepared and evaluated for their mesophase behavior. The synthesized series constitutes five members that possess different terminally attached polar groups (X). Their molecular structures were confirmed by elemental analyses and both FT-IR and NMR spectroscopy. Examination of the prepared derivatives was conducted via experimental and theoretical tools. Mesomorphic investigations were carried by polarized optical microscopy (POM) and differential scanning calorimetry (DSC). DSC and POM measurements indicated that except for the un-substituted analogue, all other derivatives were purely nematogenic, possessing their nematic (N) mesophase enantiotropically. This is to say that insertions of terminal polar substituents on their mesogenic structures induced the N phase. In addition, the location of lateral and terminal polar moieties played a considerable role in achieving good thermal N stability. Computational calculations were investigated to determine the deduced optimized molecular structures. Theoretical data indicated that both size and polarity of the terminal substituent (X) have essential impact on the thermal parameters and optical properties of possible geometries.

## 1. Introduction

The thermal stability of azobenzenes and the possibility for their molecular-mobility in response to light and heat make them suitable for many photonic applications [1,2,3,4,5,6]. Additionally, their rigidity and linear geometry make them ready to exhibit liquid crystalline phenomena [7,8]. They can also produce photoactive mesomorphic materials, whereby they can easily undergo photo-induced trans/cis isomerization. Among many mesogenic linkages, azobenzenes are the most widely documented [9,10,11,12,13,14,15,16,17,18,19,20].

Changing the core structure or insertion of lateral substituents to azobenzene-based molecules affects marked differences in their photophysical and thermal properties [6,7]. The introduction of lateral groups with different volumes and polarities broadly improves many properties of liquid crystal (LC) materials. This is attributed to the disturbance in the molecular packing which drops the melting transition and thermal mesophase stability of LCs [21,22,23,24,25,26,27,28]. Anisotropic molecules are produced from the overall molecular geometry of architectures and the combination of both rigid and flexible chains. On the other hand, as the terminal flexible chain length increases, the molecules tend to be oriented in parallel arrangements [29]. Modification of the behaviors of LCs will impact the mesomorphism and their characteristics considered essential for many technological fields. Moreover, their lateral or terminal polar group significantly affects the mesomeric characters of the azobenzene derivatives.

Today, computational quantum chemistry of newly constructed materials has attracted attention for its many potential applications [30,31,32,33,34,35,36,37,38,39,40,41,42,43]. The DFT method is a good performance theoretical tool and gives excellent computational results. The mutual enhancement of many geometrical parameters requires predicted information of their molecular orbital energies. DFT calculations will provide a prediction of molecular geometry in the gas phase. Thus the presence of the molecule in a condensed mesophase will lead to a slight difference in the resulting energy. Consequently, more elongated species will be preferred [38,44].

Insertion of the F-atom, as a lateral group, offers new mesomorphic behavior for the investigated LC derivatives [45,46,47,48] which is mainly dependent on the position and orientation of the F-atom. The high polarity and small volume of the F-atom enables it to enhance the mesomorphic and geometrical properties of the prepared LC compound such as its melting point, mesophase transitions, dipole moments, and dielectric anisotropy [49,50,51,52].

Recently, a homologous series possessing lateral F moiety [48] was synthesized and their mesomorphic behavior was investigated. These investigated derivatives and their two terminal wings are of flexible long chains. These compounds were found to exhibit N mesophases, and the position of F-atom in the molecular structure center was found to influence the physical and geometrical parameters of the molecules. In other studies [53,54,55,56,57,58,59,60,61], the investigation was directed toward measuring the impact of incorporation of lateral moiety on their mesomorphic properties in the terminal or central benzene rings.

**Scheme 1:**
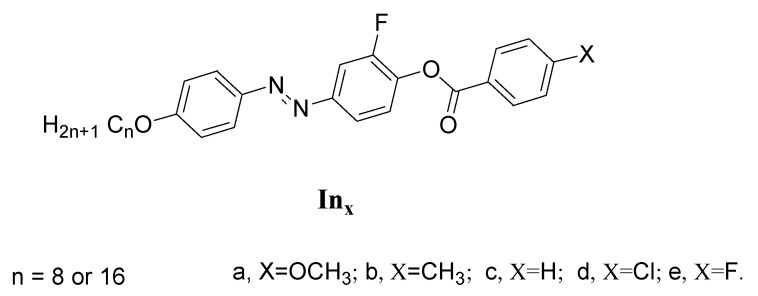
Homologous series, **In_x_**.

The goal of the present study is to focus on synthesizing new azo/ester liquid crystalline systems possessing a lateral F-atom in their central cores and unsymmetrical terminals, namely 2-fluoro-4-((4-(alkyloxy)phenyl)diazenyl)phenyl 4-fluorobenzoate (**In_x_**, Scheme 1), and to discuss their mesomorphic and geometrical parameters. We also investigate the effect of the location of the lateral F-atom and the length of terminal alkoxy chain (as well as the other different terminal compact groups) on the type and the stability of the formed mesophase. This study also aims to investigate the effect of the predicted computational DFT parameters on the optimized structures and determine how these parameters could affect their mesomorphic properties.

## 2. Experimental

### Synthesis of 2-Fluoro-4-((4-(alkyloxy)phenyl)diazenyl)phenyl 4-Substitutedbenzoate **In_x_**

**Scheme 2:**
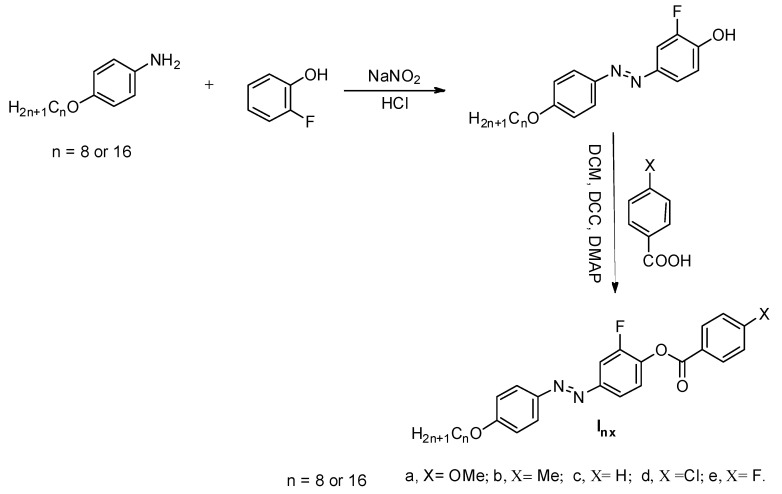
Synthesis of 2-fluoro-4-((4-(alkyloxy)phenyl)diazenyl)phenyl 4-substituted benzoate **In_x_**.

The mesomorphic compounds **In_x_** were synthesized according to Scheme 2 and all spectra of derivatives are depicted in Figures S1–S6 (Supplementary data):

## 3. Results and Discussion

### 3.1. Mesomorphic and Optical Properties

**Figure 1:**
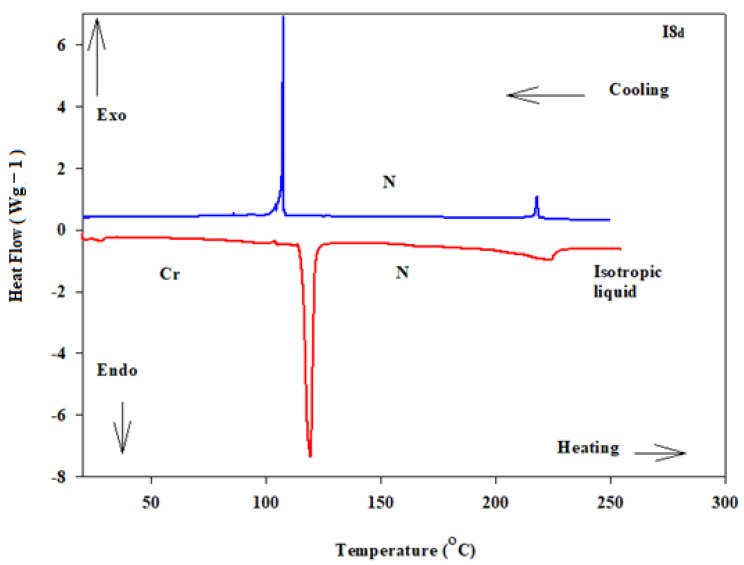
DSC thermograms captured from the second heating/cooling scan at heating rate 10 °C/min of compound **I8_d_**.

**Figure 2:**
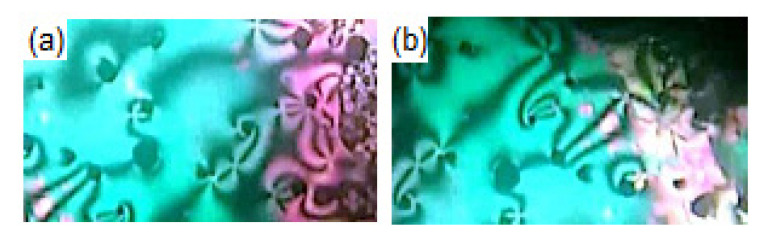
Images (320 × 97 px) of N phases under POM observed on heating for compounds (**a**) **I8_d_** at 190.0 °C and (**b**) **I16_b_** at 120.0 °C.

**Figure 3:**
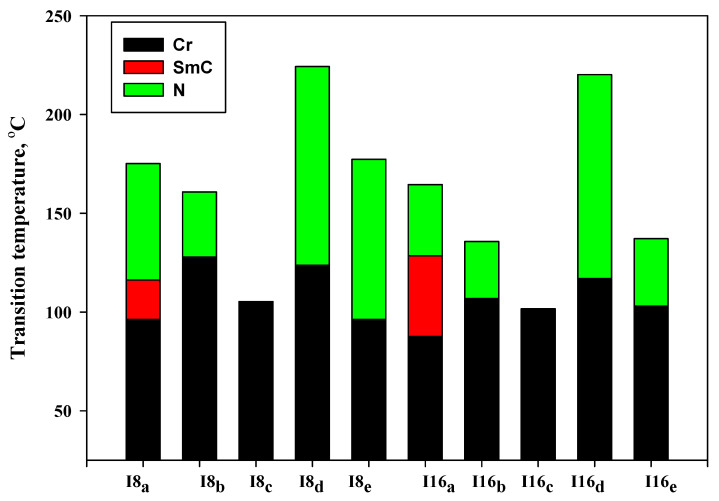
Effect of terminal (X) on the phase behavior of the investigated series **In_x_**.

Details of the mesophase transitions of all of the evaluated homologues **In_x_** series, as measured via DSC, are collected in Table 1. The types of phases, identified by POM for the present homologues, are in agreement with their DSC results. Figure 1, a representative example, depicted the DSC thermogram of **I8_d_**, taken as second heating/cooling rounds. The N phase schlieren textures of compounds **I8_d_** and **I16_b_** observed under POM are displayed in Figure 2. Phase transition temperatures of all of the derivatives are graphically displayed in Figure 3 in order to investigate the effect of terminal alkoxy-chain length (*n* = 8 and 16) on the mesomorphic properties in each group bearing different terminal polar substituent X.

The results of Table 1 and Figure 3 revealed that the melting transitions of both homologues (**I8_x_** and **I16_x_**) have irregular trends. Melting temperature depends on the polarizability of the molecule as well as its geometrical structure. Moreover, all the compounds of the homologous series are enantiotropic, exhibiting mesophase thermal stability and mesomorphic temperature range depending on their terminal group X, except for the un-substituted derivatives (**I8_c_** and **I16_c_**) which show non-mesomorphic behaviors. In case of the electron-donating terminal methoxy substituted homologues (**In_a_**), both compounds are dimorphic possessing enantiotropic SmC and N mesophases. The SmC range (Δ*T*_SmC_ = *T*_SmC_ − *T*_cr_) increases from 19.9–40.9 °C with increasing the length of terminal alkoxy chain from *n* = 8 to *n* = 16, while the nematic range (Δ*T*_N_ = *T*_iso_ − *T*_SmC_) is dropped from 59.0–36.0 °C as the alkoxy chain length increases from 8 to 16 carbons. It was documented that the nematic thermal stability decreases with *n* [62,63]. Thus, the derivative of shortest terminal-chain (**I8_a_**) exhibits a higher N thermal stability value (175.2 °C) than the higher homologue **I16_a_**, which possesses an N stability value of about (164.5 °C). The two homologues possess smectic C stabilities 116.2 and 128.5 °C for **I8_a_** and **I16_a_**, respectively. Homologues with the terminal CH_3_ group (**In_b_**) are found to be monomorphic, possessing only the N phase. On the other hand, Δ*T*_N_ is decreased from 32.9–28.8 °C with an increasing *n* from 8 to 16. Additionally, the thermal N stability is reduced from 160.8–135.7 °C as the terminal length increases from *n* = 8 to *n* = 16. In the case of the unsubstituted homologues (X = H, **In_c_**), all of the homologues exhibit non-mesomorphic behaviors, irrespective of their terminal alkoxy chain length. Moreover, only one endotherms characteristic peak of the crystal–to–isotropic transitions was observed in DSC measurements upon heating and reversed during the cooling cycle for **In_c_**. In addition, the POM investigations showed no mesophase textures, affirming that the materials do not exhibit any mesomorphic properties.

Electron-withdrawing terminally substituted Cl homologues (**In_d_**) showed the broadest nematic temperature range and stability. The N phase stability and temperature range for **I8_d_** reached values of 224.3 and 100.6 °C, respectively. By increasing the length of the terminal chain to *n* = 16 (**I16_d_**), the N phase stability was slightly reduced to 220.2 °C and the nematogenic temperature range became 103.3 °C. In the case of the small size terminal F-atom derivatives (**In_e_**), the compounds were also purely nematogenic and exhibited only the N mesophase. Moreover, Δ*T*_N_ was reduced from 81.0 to 34 °C and the N stability was reduced from 177.3 to 137.1 °C with an increase of *n* from 8 to 16 carbons. The descending trend in the N thermal stability can be ascribed to the dilution of the rigid mesogenic portion.

Based on the above results, the thermal N phase stability declined in the following order: Cl > F > OCH_3_ > CH_3_ for **I8_x_**, and Cl > OCH_3_ > F > CH_3_ for **I16_x_**, while the un-substituted homologues (**In_c_**) were non-mesomorphic. Thus, the introduction of the terminal polar group induced the mesophase when incorporated in the mesogenic structure. In general, the polarity of the attached-groups, aspect-ratio, polarizability, molecular rigidity, and shape of the molecule are considered to be essential factors responsible for the thermal stability and range of the formed mesophases and their texture types. The factors that affect the mesophase properties will be briefly discussed in the next section of theoretical calculation studies.

The normalized entropy changes of mesophase transitions (Δ*S*/R) for all designed laterally F members (**In_x_**) are collected in Table 1. Data indicate that, independent of the terminal substituents, the N-I entropy transitions showed irregular trend and lower values. Their relatively small magnitudes may be due to the somewhat molecular biaxiality and the relatively high values of clearing temperatures, which in turn reduced the N-I entropy changes [64,65,66]. The configuration of the lateral F-atom plays a considerable role in the physical and geometrical parameters of the molecule. This will be briefly discussed in the theoretical work of this study. Additionally, the azo moiety thermal cis/trans isomerization leads to lower entropy changes, as has been determined before (for example, see [65,67,68,69]).

### 3.2. Computational Calculations

#### 3.2.1. Thermal and Geometrical Parameters

**Figure 4:**
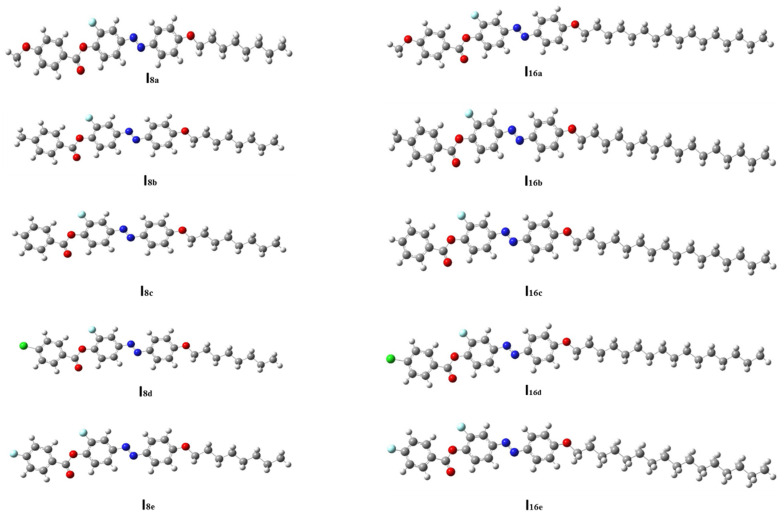
The optimized molecular structures of analogues series **In_x_**, predicted at B3LYP/6–311G ** level.

The geometry of a compound is an inherent property which dictates other properties showcased by the molecule. Based on this, the optimized geometrical structure of each member of the present series (**In_x_**) was determined in order to establish the most stable geometry for the molecule. The results are presented in Figure 4. Theoretical calculations were carried out via the DFT method for all of the designed derivatives in order to correlate the predated quantum chemical parameters and the experimental findings. Computational calculations were performed in the gas phase with DFT/B3LYP program at basis set 6–311G **. The designed homologues (**In_x_**) ere proved to exist in planar conformation since they possessed mesomorphic characters, except for the unsubstituted derivatives which are non-mesomorphic. Zero-point energy and other predicted thermal parameters are summarized in Table 2 and Table 3. It was found that all of the estimated parameters increased with an increasing of the terminal alkoxy-chain length from *n* = 8 to *n* = 16. Moreover, these parameters normally changed according to the type of the terminal group (X). All compounds show linear and planar shapes, as displayed in Figure 4. It was documented in [70] that the mesogenic core planarity of the mesomorphic molecules is influenced by the mesomeric-nature of the polar attached moieties. Thus, the conjugated π-cloud interactions resulting from the polar lateral F and terminal X groups played a considerable role in inducing mesomorphic phenomena with good physical parameters.

As shown in Table 3, the lower magnitudes of predicted ionization potential (I.E) for the **In_a_** compounds indicate a more basic nature of the terminal OCH_3_ derivatives than other compounds in the prepared series [71]. Additionally, a pronounced increase of polarizability is observed for member **I16_a_** that may be due to the increment of the molecular shape aspect ratio. In general, the polarity of the attached substituents, rigidity and polarizability as well as geometry of the mesomorphic molecule are considered important parameters for inducing and influencing the mesophase behavior. In addition, the thermal stability is mainly dependent on the length of the terminal chains [72].

#### 3.2.2. Frontier Molecular Orbitals (FMOs)

**Figure 5:**
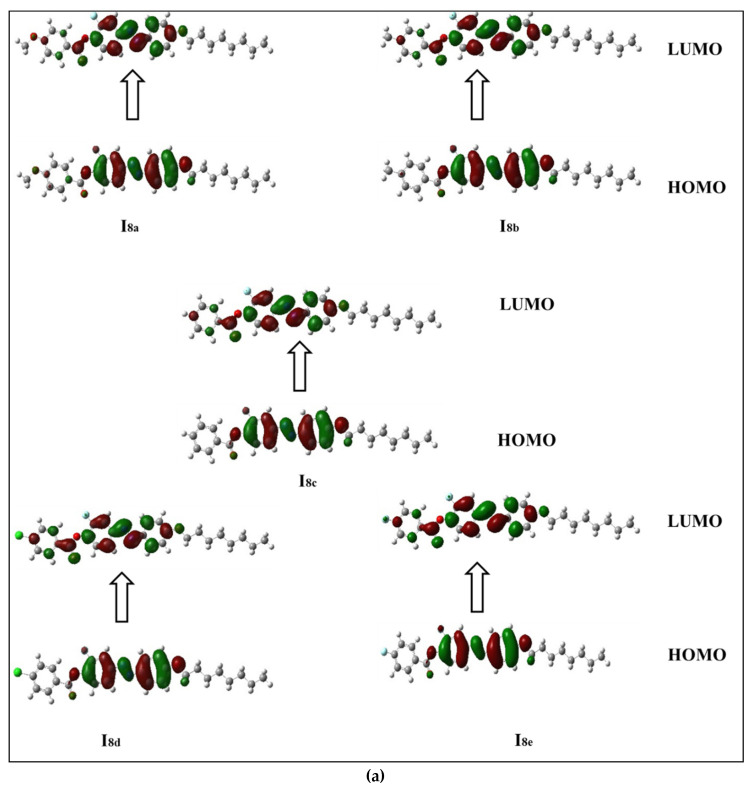

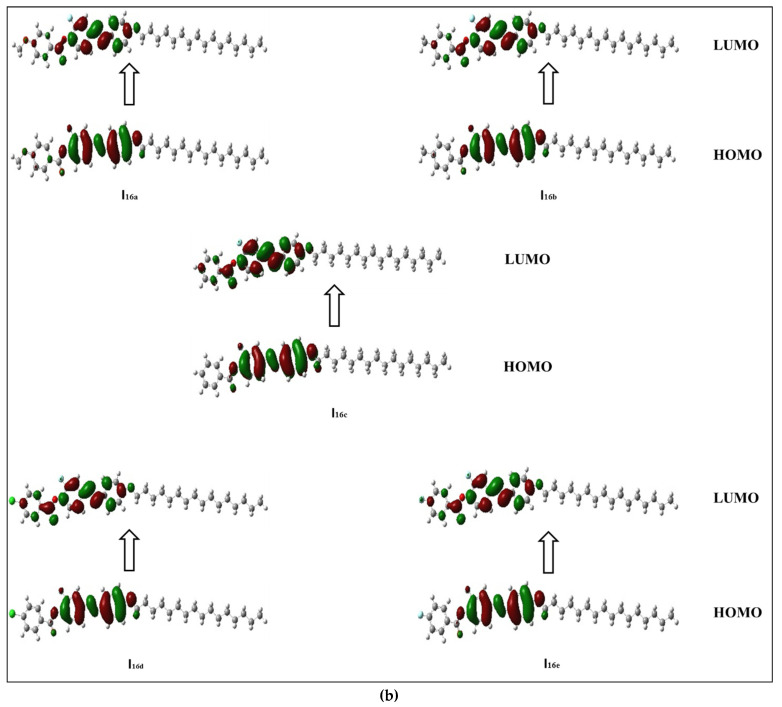
Estimated FMOs for of the synthesized analogues sets (**a**) **I8_x_** and (**b**) **I16_x_**.

The frontier molecular orbitals presented in Figure 5a,b show that all compounds in the series (**In_x_**) have different HOMO and LUMO distributions. For the HOMO, the electron clouds were evenly distributed over the carbon atoms and the π-electron of the two benzene rings bearing the lateral F, as well as the linking groups. On the other hand, the LUMO distributions of electron clouds are extended to the third phenyl ring bearing the alkoxy chain. The resulting energies and energy gaps for the present investigated **In_x_** series are collected in Table 3. The HOMO and LUMO energy gap (∆E) are both related to the chemical reactivity of the compounds. More reactive molecules have an energy gap with a lower magnitude. Predicted ∆E (Table 3) confirms that the **In_d_** derivatives (X = Cl) are more reactive than the other compounds. They are also softer than other members due to that fact that the ∆E is inversely related to the softness. Furthermore, the FMO energy gaps are highly impacted by the orientation and location of the mesogens (ester and azo linkages). This orientation cloud allows for the maximum delocalization of the 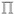 -electrons, and thereby decreases the ∆E of the FMOs. 

#### 3.2.3. Molecular Electrostatic Potential (MEP)

**Figure 6:**
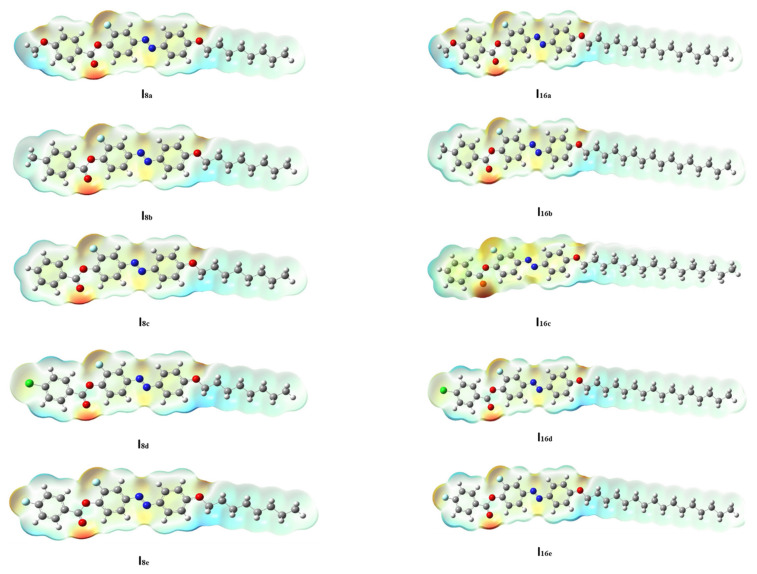
MEP of series, **In_x_**.

The geometry of the prepared mesomorphic molecules was impacted by the mesomeric configurations, which were in turn affected by molecular-molecular interactions. The polarizability, electronic shape, dipole moment, and other parameters were impacted by the distribution of the electron density at the atomic sites of the liquid crystalline compounds [73]. MEP is considered to be one of the best tools for the estimation of the presence of inter-/intra-molecular interactions the evaluated molecules. The MEP of the present series (**In_x_**) are shown in the Figure 6. Here, the shadowing of carbonyl oxygen and the lateral F-atom by a red cloud suggests a low electrostatic potential but a high electron density for these regions. On the other hand, the blue cloud over the first alkoxy methylene and the neighboring phenyl hydrogen indicates low electron density but high electrostatic potential. Additionally, as the terminal chain length increases from *n* = 8 to *n* = 16, the polarizability of the molecule increases. This observation could be explained in the term of the less ordered nematic phase which covers all of the prepared molecules of terminal polar groups. Additionally, Figure 6 indicated that the location of mesogens and the electronic nature of the terminal polar X influenced the distribution of the MEP. Moreover, they appeared to impact the mesophase kind and stability by altering the competitive forces between end-end and side-side interactions. In recent studies, we have investigated the correlation between computational charge distribution and mesomorphic properties [48,60,74,75]. The increment of the charge distribution on the molecules is attributed either to the greater electron-donation or electron-acceptance of terminal aggregations to enhance the N phase or the parallel-interactions to predominate the semectic phase.

## 4. Conclusions

New fluorinated liquid crystalline analogous series possessing different terminal polar compact groups, namely, 2-fluoro-4-((4-(alkyloxy)phenyl)diazenyl)phenyl 4-substitutedbenzoate, were prepared and investigated for their properties via experimental and computational measurements. Their molecular structures were determined via different spectroscopic analyses. Both mesomorphic and optical examinations were measured using DSC and POM and revealed that all of the designed analogues are monomorphic and purely nematogenic with enantiotropic properties (except for the un-substituted analogues, which demonstrated non-mesomorphic behavior). The selected terminal polar electron donating and electron withdrawing groups contributed to achieving the induced N phase with good thermal stability. DFT theoretical calculations revealed that the lateral and terminal polar groups have essential effects on the stability of the predicted geometries and their thermal parameters. Moreover, the produced N mesophase was dependent on the influenced dipole moment of the mesogenic portion, which was in turn dependent on the geometrical shape of molecule.

## Figures and Tables

**Table 1:** Mesomorphic temperature of transitions (***T*** °C), enthalpy of transition Δ*H*, kJ/mole, and normalized entropy of transition Δ*S*/R for investigated set, **In_x_**.

Comp	*X*	*T*_Cr-I_	Δ*H*_Cr-I_	*T*_Cr-SmC_	Δ*H*_Cr-SmC_	*T*_Cr-N_	Δ*H*_Cr-N_	*T*_SmC-N_	Δ*H*_SmC-N_	*T*_N-I_	Δ*H*_N-I_	Δ*S*/R
**I8_a_**	-OCH_3_	-	-	96.3	45.15	-	-	116.2	2.80	175.2	1.94	0.52
**I8_b_**	-CH_3_	-	-	-	-	127.9	51.76	-	-	160.8	2.13	0.59
**I8_c_**	-H	105.3	57.64	-	-	-	-	-	-	-	-	-
**I8_d_**	-Cl	-	-	-	-	123.7	46.70	-	-	224.3	2.09	0.51
**I8_e_**	-F	-	-	-	-	96.3	39.38	-	-	177.3	1.23	0.33
**I16_a_**	-OCH_3_	-	-	87.6	48.78	-	-	128.5	2.90	164.5	1.48	0.41
**I16_b_**	-CH_3_	-	-	-	-	106.9	44.6	-	-	135.7	1.62	0.48
**I16_c_**	-H	101.6	59.87	-	-	-	-	-	-	-	-	-
**I16_d_**	-Cl	-	-	-	-	116.9	46.32	-	-	220.2	1.87	0.46
**I16_e_**	-F	-	-	-	-	103.1	42.30	-	-	137.1	1.36	0.40

Cr-N refers to the solid-to-nematic phase. N-I refers to the nematic-to-isotropic liquid phase.

**Table 2:** Estimated thermal parameters for present series, **In_x_**.

Compound	ZPE (Kcal/Mol)	Thermal Energy (Kcal/Mol)	Enthalpy (Kcal/Mol)	Gibbs Free Energy (Kcal/Mol)	Entropy (Cal mol·k)
**I8_a_**	339.931	360.875	361.467	295.216	222.206
**I8_b_**	336.987	358.034	358.627	290.124	229.761
**I8_c_**	319.853	339.674	340.266	275.311	217.862
**I8_d_**	313.578	334.225	334.818	267.740	224.983
**I8_e_**	314.447	334.832	335.424	269.207	222.091
**I16_a_**	483.249	511.573	512.165	426.784	286.371
**I16_b_**	480.291	507.554	508.147	425.184	278.260
**I16_c_**	463.181	489.788	490.381	408.976	273.032
**I16_d_**	456.900	484.336	484.928	401.334	280.376
**I16_e_**	457.772	484.943	485.536	402.825	277.414

**Table 3:** Total Energy, E_HOMO_, E_LUMO_, ∆E, dipole moment, ionization energy, electron affinity, and polarizability for the investigated series, **In_x_**.

Compound	Total Energy (Ha)	E_HOMO_ (ev)	E_luMO_ (ev)	∆E (ev)	Dipole Moment (D)	IE (ev)	EA (ev)	Polarizability Bohr^3^
**I8_a_**	−1595.223	−6.043	−2.515	3.528	2.6252	6.043	2.515	424.26
**I8_b_**	−1520.026	−6.074	−2.547	3.527	1.8676	6.074	2.547	414.42
**I8_c_**	−1480.739	−6.108	−2.586	3.522	2.6458	6.108	2.586	396.84
**I8_d_**	−1940.352	−6.194	−2.701	3.493	5.4948	6.194	2.701	414.23
**I8_e_**	−1579.994	−6.175	−2.668	3.507	5.0740	6.175	2.668	397.89
**I16_a_**	−1909.479	−6.042	−2.515	3.527	2.6315	6.042	2.515	519.56
**I16_b_**	−1834.285	−6.074	−2.547	3.527	1.9022	6.074	2.547	509.61
**I16_c_**	−1794.997	−6.107	−2.586	3.521	2.6949	6.107	2.586	492.04
**I16_d_**	−2254.610	−6.193	−2.701	3.492	5.5510	6.193	2.701	509.50
**I16_e_**	−1894.251	−6.174	−2.667	3.507	5.1293	6.174	2.667	493.10

## Data Availability

Not applicable.

## Supplementary Materials

The following are available online. Figure S1–S6: Materials, Methods and spectroscopic analyses, Characterizations.
